# Strategic concept paper for bioeconomy in Poland: executive summary

**DOI:** 10.12688/openreseurope.16229.1

**Published:** 2023-12-14

**Authors:** Jerzy Kozyra, Paweł Chmieliński, Piotr Jurga, Mariusz Maciejczak, Magdalena Borzęcka, Justyna Cieślikowska, Stelios Rozakis

**Affiliations:** 1 Bioeconomy and Systems Analysis, Institute of Soil Science and Plant Cultivation – State Research Institute, Puławy, 24-100, Poland; 2 Department of European Integration, Institute of Rural and Agricultural Development of the Polish Academy of Sciences, Warszawa, 00-330, Poland; 3 European Rural Development Network (ERDN), Milanówek, 05-822, Poland; 4 Institute of Economics and Finance, Warsaw University of Life Sciences – SGGW, Warszawa, 02-787, Poland; 5 Department of Innovation, Digitalization and Knowledge Transfer, Ministry of Agriculture and Rural Development, Warszawa, 00-930, Poland; 6 School of Chemical and Environmental Engineering, Technical University of Crete, Chania Crete, 73100, Greece

**Keywords:** Sustainable circular bioeconomy, strategic options, transformation pathways, organic farming, fresh water aquaculture, biogas and biomethan

## Abstract

The main objective of the proposed approach is to accelerate the transition of the bioeconomy towards a knowledge-based sustainable system, covering key biobased sectors strongly linked to agriculture in Poland, in line with the European Green Deal. The proposed model of a bioeconomy development strategy, with a special focus on agriculture, is based on two pillars: (1) strengthening traditional, relevant (in terms of economic indicators) sectors of the economy and improving their ‘sustainability’ by implementing the proposed transformation pathways; and (2) developing economic activities or ‘niche or novel sectors’ that are prospective accelerators of change in the face of climate challenge, and ensuring their upscaling. This approach forms the basis for policy planning at national and regional level in these European Union (EU) countries, where bioeconomy development strategies have been initiated through dialogue between science, administration and industry stakeholders. The strategic actions we propose are grouped in three areas; (1) Market intervention mainly by introducing sustainability criteria for the national production system; (2) Research, innovation and education that significantly strengthen the relationship between business and science and educational activities in the field of sustainability and climate change, and (3) Governance and policy actions to enforce the relationship between the main sub-sectors of bioeconomy and niche sectors in frame of bio-economy strategy or action plan that will profits by added value of products from bioeconomy sectors and increasing number of newly created jobs.

## Introduction

The consequences of climate change over the last decade show that without careful planning of the use of available bioresources the country's viable future will be threatened. The circular sustainable bioeconomy could provide climate-neutral solutions and become the core of the sustainable model for resilient economies ( Rozakis *et al.,* 2024). To achieve this, a cross-sectoral approach to the production and processing of bioresources and strengthened strategic planning are required. Strategic thinking on the sustainable use of bioresources at national and macro-regional level should become a priority in order to ensure food, energy and industrial security.

The BIOEAST initiative was established by declaration of the ministers of agriculture of Visegrad Group and Bulgaria, Romania and Slovenia during the meeting on 25–26 October 2016 in Warsaw for the stronger inclusion of the research potential of the Central and Eastern European (EU-13) countries for building knowledge-based agriculture, forestry and aquaculture in the bioeconomy. To support this idea, presented in the BIOEAST Initiative Vision Paper, the BIOEASTsUP project (Horizon 2020 EU) has been launched to develop the blueprint for bioeconomy strategies in each of the eleven partner countries. The conceptual study on the directions of bioeconomy development in Poland can serve as a collection of postulates developed by research centers for policy makers at the national and regional level during the implementation of the BIOEASTsUP project implementation, which are described among others in detail in the ( Dach *et al.,* 2022; Kulišić *et al.,* 2020; Rozakis *et al.,* 2023; Vitunskienė *et al.,* 2021). This paper is intended for discussion by policy makers and stakeholders in the process of building bioeconomy strategy using BIOEASTsUP project achievements. It attempts to provide a coherent vision of a strategic approach to address the problem of the adverse impacts of human interaction with nature, or rather to integrate these relationships into a holistic (and systemic) view of the Earth system as a community of resources, values and rights. We emphasize the need to first ensure the sustainable development of bio-based sectors, on which the concept of bioeconomy imposes a double burden of providing food for a growing population and biomass to be used for products and processes. The main objective of the proposed approach is to initiate the bioeconomy transition of the bioeconomy to a knowledge-based sustainable system covering the key bio-based sectors, strongly linked to agriculture in line with the European Green Deal.

## The concept of a systemic approach to bioeconomy

The definition of the bioeconomy has been evolving over the years, so that in recent period the focus on sustainable use of biological resources predominates ( Maciejczak & Hofreiter, 2013). As a result, the actual characterization of the bioeconomy emphasizes the aspect of sustainability and circularity. According to the latest report of the European Commission, bioeconomy policy should be based on all three dimensions of sustainable development, namely environmental, economic and social ones respectively specified as: (1) management of land and biological resources, taking into account the ecosystem boundaries; (2) sustainability of value and consumption chains; and (3) the pursuit of social justice and just transitions ( European Commission, 2022). The circular economy is a concept aiming at the rational use of resources, according to which materials and products should remain in the economy as long as possible, and the generation of waste should be kept to the minimum. The definition of bioeconomy formulated in the Roadmap for the Circular Economy (CE) in Poland ( Polish Council of Ministers, 2019) indicates that bioeconomy is a closed-loop economy i.e., a biological cycle in the economy is one of the two pillars of the CE, alongside the technological cycle. The biological cycle in CE is related to the management of renewable resources – the so-called biomass – throughout its life cycle. This includes production of agricultural raw materials, production of goods (e.g., food, feed, bioenergy), processing, sale of goods, the use phase, and bio-waste management.

The Polish National CAP Strategic Plan (2022) indicates the necessity to pay special attention to the biomass and production of bio-based products. The bioeconomy sector in Poland in 2019 was responsible for 14% of employment and it generates an annual value of 147 billion Euro ( Tamošiunas *et al.,* 2022). The data show that the largest share in the bioeconomy turnover in the EU and in Poland is represented by the food, feed, and beverage production sectors, which account for nearly half of the total turnover. The turnover of bioproducts, including the production of chemicals and chemical products, pharmaceuticals, plastics, paper, textiles, bio-fuels and bio-energy, and the wood industry sector, is worth around 690 billion Euro – according to Com-mission’s platform for management and analysis of data and the output from models addressing issues of resource economics and sustainability (Data M): for these sectors throughout European Union. The agricultural and forestry sector is mainly responsible for the production of raw material for the biomass processing industry. Agricultural products generate 17% and wood products 4% of the total product value in the economy with notable regional differentiation regarding input-output coefficients ( Jurga *et al.,* 2021).

The most relevant paths for the transformation of the current fossil-based economy to a sustainable circular bioeconomy system comprise the following directions: (1) sustainable intensification of the productivity of the primary sector; (2) replacement of fossil fuels in energy production and increasing the share of energy from renewable sources; (3) new or more efficient biomass use streams; (4) increasing the use of high value added intermediates; (5) valorization (monetization) of ecosystem services ( Dietz *et al.,* 2018).

The transition to sustainable agriculture, forestry and fisheries in the bioeconomy does requires specific sustainability conditions and adoption of sector-specific transformation pathways. The European Foresight Exercise developed by the Standing Committee of Agricultural Research ( Mathijs *et al.,* 2015) identified forth the five principles of the sustainable bioeconomy: food first, sustainable yields, cascading approaches, circularity and diversity should be strived for. The transition cannot be driven by markets and technology, but strong strategic orientation and constant monitoring is necessary. The premises of the transition should be enabled by principles and political willingness. This approach constitutes the basis for policy planning at the national and regional level in these EU countries where bioeconomy development strategies were triggered by a dialogue between science and administration as well as industry stakeholders. The transition capacities can be detected by a system approach to bioeconomy ( Maciejczak, 2017). Considering main system components namely renewable resources, products and services, knowledge, innovations and technologies, research and development, private and public expectations, as well as the pivot component ‘finance and governance’ the analyst can derive driving and constraining factors for transition. Based on these factors strategic analysis has detailed internal and external strengths and weaknesses for the deployment of bioeconomy in Poland shown briefly in the following paragraphs.

## Strengths of the bioeconomy in Poland

Poland is one of the EU countries with a high potential in the production and use of biomass ( Loizou *et al.*, 2019) and resulting waste biomass resources ( Hamelin *et al.,* 2019), with the prospect of further development to supply the internal and external markets. Based on the volume of cited publications in world scientific literature, agricultural and biological sciences as well as biochemistry, genetics and molecular biology are the most studied topics in Poland. The country has a significant potential of agricultural knowledge and innovation systems (AKIS) with public extension services and research, development, and innovation (RDI) and educational institutions at disposal: 10 agricultural universities, seven universities educating in the field of forestry, two universities educating in fields related to fisheries, 12 agricultural research institutes, one forestry research institute, two research institutes dealing with fields related to fisheries, and 24 institutes dealing with bioproducts and biomass processing (Łukasiewicz Research Network). Poland has significant potential to educate in bioeconomy in frame of secondary level schools in the field of agriculture (62 schools) and forestry (11 schools), supporting bioeconomy extension and knowledge transfer. Lessons learned so far from implementation of innovations by sectors such as agriculture, food, forestry and renewable energy, can be used both to absorb innovations and to adapt to the new challenges of the bioeconomy ( Chaber *et al.,* 2022).

## Weaknesses of the bioeconomy in Poland

According to expert opinions, gathered during the BIOEASTsUP project interaction panels, the main weakness of polish bioeconomy sectors is insufficient knowledge about the level of sustainability of the products, including the carbon footprint and circularity, which makes it difficult to coordinate work towards achieving climate neutrality in Poland. There is also a lack of recognition of the relationships between economic sectors in bioeconomy value chain ( Loizou *et al.,* 2019). Despite the high potential for RDI development, the level of interaction between bio-based industry and academia is still low, and even lower in the agricultural sector than in other sectors. Only one in five companies report interaction and cooperation with research centers (except in the pharmaceutical sector). The number of national patents for bioeconomy technologies is very low ( Vitunskienė *et al.,* 2021). Another weakness is the low average productivity of farms in Poland, compared to the EU, which is a result of agricultural fragmentation, lower soil quality and a relatively shorter growing season than in most of the European countries ( Chmieliński & Wieliczko, 2022). The labour productivity in bioeconomy sectors in Poland is below the EU-27 average ( Kulišić *et al.,* 2020) and the Polish economy is highly dependent on the global value chains based on the use of foreign inputs in exports (reverse participation) and the provision of intermediate goods and services that are further used in the exports to other countries ( Kulišić *et al.,* 2020). There are still underdeveloped and insufficiently integrated prospective bioeconomy sectors, such as: biogas and biomethane sector (use of heat, fertilizing products for the circular economy) ( Dach *et al.,* 2022), and inland freshwater aquaculture ( Rozakis *et al.,* 2023), with underdeveloped bioeconomy clusters (business support institutions) ( Vitunskienė *et al.,* 2021).

## How to develop bioeconomy sectors to ensure sustainable development - which sectors require intervention and which are the growth drivers?

Based on the work carried out within the BIOEASTsUP project, literature reviews, analyses of available data carried out by the expert networks, thematic working groups of the BIOEAST initiative, and consultations at the level of national working groups, it has been possible to identify measures that should serve as drivers for the bioeconomy deployment in two areas that require different frameworks: (1) conventional sectors including agriculture, timber and food production and (2) niche sectors with case studies for organic agriculture, agro-energy: biogas and biomethane, aquaculture. One of the main objectives recognized behind the development of the Polish Bioeconomy Strategy is to create an ecosystem that can support the sustainable transition to a climate-neutral future and add value to the locally available bio-resources. The ultimate goal should be creation value-added processes that contribute to raising the standard living of Polish society.

### Conventional bioeconomy sectors

Conventional bioeconomy sectors in Poland include agriculture and forestry, as biomass production sectors, and those with the food-industry, which all constitute the most important part of the Polish Bioeconomy, thus they are representing the main challenge in the sustainable transition. The productivity of farms in Poland is one of the lowest in the EU as a result of agrarian fragmentation, as well as lower soil quality and a shorter growing season than in Western European countries ( Pawlak & Poczta, 2020). In such a situation, the need to reduce the use of mineral fertilizers and plant protection products postulates by European Green Deal is linked to the need to implement technological progress and improve knowledge and skills in the biomass production and food/feed processing sector. Therefore, the conventional bioeconomy sectors require better regional planning coordination towards: (1) the review of production technologies to increase their productivity: including the exploitation of marginal and abandoned land, reducing the use of natural resources and increasing the use of biobased fertilizers; (2) reconsidering the biomass value-chains by overarching systemic management of agricultural biomass to fully exploit its economic potential for both food and non-food use, i.e., food and non-food valorization should be connected by promoting the interconnection of production and processing sectors of the bioeconomy (including production of biomaterials, biomethane, etc.); (3) developing monitoring systems to monitor the carbon footprint of processes and products; (4) strengthening the relationship between the main sub-sectors of the food sector, the biogas and biomethane sectors, and the production of bioproducts and bio-based carbon as well.

In order to accelerate the transformation process, it is necessary to undertake the following actions in conventional bioeconomy sectors: (1) significantly strengthen the relationship between business, science and educational activities in the field of sustainability and climate change, as well as in the search for novel technological, organizational and social solutions; (2) bring about a significant reduction in the level of inputs and costs of the use of plant protection products and fertilizers; important investments are necessary, as well as an appropriately large scale of production on farms and the use of modern techniques and technologies, which will allow for compensating the reduction of the applied doses of fertilizers and plant protection products with higher effectiveness of their impact (i.e. precision farming), as well as ‘regenerative’ farming (inter alia, no-till, digital maps of soil and crop abundance, monitoring, application of measures depending on the needs of plants, etc.); (3) implement a system for data collection, assessment, monitoring and management of biomass for the food chain and for non-agricultural use in bioeconomy sectors; (4), implement a system to assess the carbon footprint of products and define actions to achieve climate neutrality in the agricultural sector and at the supply chain level, to enhance the competitiveness of agriculture; (5) create sustainable relation-ships between key sub-sectors of the food sector, the biogas and biomethane sector and the production of bio-based products; (6) carry out systematic educational activities regarding sustainability and climate change, waste management, responsible consumption and healthy diet.

### Niche bioeconomy sectors

A strengths, weaknesses, opportunities, and threats (SWOT) analysis of bioeconomy sectors indicated identified by experts pointed three niche sectors of Polish bioeconomy that need to be primarily taken into account which are: organic farming, biogas and biomethane, and inland aquaculture ( Vitunskienė *et al.,* 2021).

Development of the organic farming sector as a niche sector in Poland, should significantly increase its impact on the overall economy, according prioritization done by the experts panels should include: (1) increasing the number of producers and consumers of organic farming products; (2) changing the perception of consumers in assessing the value of organic farming products as high value added products; (3) increasing technical capabilities of organic farming and the availability of knowledge and dissemination of information on organic farming in Poland; and (4) increasing the possibility of interaction between producers and consumers in order to boost consumer involvement in the sustainable development of organic farming. In order to eliminate barriers to the development of the organic sector in Poland, it is necessary to: (1) increase the level of the institutional – non-financial support to farmers, through technological support, advisory services, market information; and (2) support the market mechanisms aimed at the development of value chains. In order to reduce the risk of unfavorable changes, the following are needed: (1) farms that have stronger market power, (2) processors that will primarily focus on organic production, (3) strong market surveillance, also by the non-governmental organizations; (4) efficient network of knowledge dissemination; and (5) efficient market information.

Development of the biogas and biomethane sector as a crucial sector for energy independence with high potential for the management of integrated supply chains in agriculture (by-products) and food industry to exploit waste that requires according the analyses performed the following measures: (1) stabilization of the economic and legal context for biogas and biomethane producers; (2) promotion of sustainable business models of biogas plants for waste management and for agriculture as a necessary element for recycling of waste biomass to maintain soil fertility; and (3) support for modern technical solutions, including flexible demand driven meso- and micro-installations. In order to eliminate barriers to the development of the biogas and biomethane sector, efforts should be made to change: (1) the perception that a biogas plant creates an unfavorable change in the ecosystem through negative environmental impact and creating a hardship for small communities (odor nuisance). In order to reduce the risk of unfavorable change, it is necessary to (1) implement monitoring of the processes in biogas plants regarding sustainable substrates through cooperation with specialized laboratories; and (2) introduce carbon certificates for biogas plants that take into account the avoided emissions.

Development of the fresh water aquaculture in Poland requires: (1) creating conditions for developing new business models for products with high added value; (2) creating support systems for fish farms, which will include a fee for ecosystem services provided by the sector, especially for water management and water retention, providing habitats for many valuable species and places for recreation; (3) establishing local partnerships of science, business and administration for strengthening cooperation within the sector and for educational purposes; and (4) promoting products of inland fisheries as products with high added value and low carbon footprint. In order to eliminate barriers to the development of the freshwater aquaculture, it is necessary to review the existing legal regulations in the light of the latest strategies with the aim to eliminate the provisions that constitute a hindrance. In order to reduce the risk of unfavorable changes, a monitoring system is needed that will consider the impact of the sector on the environment and will be able to present a range of values resulting from the supply of products with high added value in a short supply chain.

## Conclusions

The proposed model of the bioeconomy development strategy with special focus on agriculture is based on two pillars: (1) strengthening traditional, relevant (in term of economic indicators) sectors of the economy and improving their ‘sustainability’ by implementing the proposed transformation paths; and (2) developing economic activities or 'niche or novel sectors' that are prospective accelerators of changes in the face of global challenges that guaranties their upscaling.

Enhanced SWOT analysis for strategy formation has been implemented firstly evaluating importance of strengths and weaknesses, so that to capacitate combinations in order to attribute priorities to the strategic propositions for action for each of the studied promising sectors ( Rozakis *et al.,* 2023). Strategic directions to develop overarching national bioeconomy appropriate emanate through a synthesis of strategic actions that can be grouped in three areas:

- **Market intervention**: introducing sustainability criteria for the national production system could help the deployment of bio-based products; the high added value products and related delivered ecosystem services should be promoted; special programs to be launched to support new business models and cooperation in local/micro cluster partnerships.
- **Research, innovation and education**: research agenda as a functional part of the macro-region Strategic Research and Innovation Agenda (SRIA) with the most important elements for bioeconomy: (1) increasing the agricultural productivity through sustainable intensification; (2) cascading the use of agricultural and forest residues potential (increasing circularity) and the added value of biomass through innovative bio-products and technologies, and (3) new and modern bio-refinery technologies and products (except for sugar and starch-based bio-refineries). Significantly strengthen the relationship between business and science and educational activities in the field of sustainability and climate change.
- **Governance and policy actions:** (1) set up a Polish Bioeconomy Council to ensure long-term engagement at the national level to act as a catalyzer for interministerial coordination; (2) enforce the relationship between the main sub-sectors of bioeconomy and niche sectors in frame of bio-economy strategy or action plan; and (3) evaluate and monitor policy adoption by appropriate indicators valid for different environmental policies, e.g. sustainability indicators (carbon footprint, water footprint, energy footprint), annual expenditure on education in individual departments/sectors, added value of products from bioeconomy sectors, number of newly created jobs.
