# Supplementary material for: Strategic concept paper for bioeconomy in Poland: executive summary
Source: Open Res Eur. 2023 Dec 14;3:217. [Version 1] doi: 10.12688/openreseurope.16229.1 (PMC10933564; doi:10.12688/openreseurope.16229.1)
Supplement: Peer review history

# Reviewer report: Reviewer response for version 1

**DOI:** 10.21956/openreseurope.17519.r38133

**Published:** 2024-03-12

**Authors:** Rodrigo Salvador

**Affiliations:** 1 Technical University of Denmark, Ballerup, Denmark

This paper aims to identify how the implementation of a bioeconomy in Poland can be prompted or accelerated. The subject is worthy of investigation and it has high relevance currently. It is well written, overall, but it lacks methodological detail, and some results could be made more evident. I provide more detailed comments hereafter.

**General:**

 The paper should undergo language revision. The content can be understood, and the text is well-written overall. However, there are places where it could benefit from clarifying or polishing the language e.g., “Strategic directions to develop overarching national bioeconomy appropriate emanate through…”.

**Title:**

 Why is the paper called “executive summary”? I suggest revising the title and delete “executive summary” from it.

**Introduction:**

 Please revise the sentence “… initiate the bioeconomy transition of the bioeconomy to a …”. It is not clear what you mean with “bioeconomy transition of the bioeconomy."

 The concept of a systemic approach to bioeconomy:

 In “The transition cannot be driven by markets and technology, but strong strategic orientation and constant monitoring is necessary”. I believe “but” could be replaced with “and thus” to represent the intended meaning. Otherwise, please revise it.

 The “Strengths of the bioeconomy in Poland” and “Weaknesses of the bioeconomy in Poland” presented in the text seem to be relevant. However, while keeping the text, I believe they could be summarize in the format of bullet points in lists (one in each section) or a Table, to make it more reader-friendly.

**How to develop bioeconomy sectors… :**

 I can see the first paragraph in this section as containing the “methods” used to conduct the research. However, this needs much greater detail. Even though the paper is under the category of an “Open Letter”, this is an academic-like paper and therefore needs an adequate methodology section to ensure transparency and replicability.

 I suggest that the authors either detail the methods used to conduct the research in this part of the paper, or create a section called methodology and do it in there. Nevertheless, the methods need to be included.

Where applicable, are recommendations and next steps explained clearly for others to follow? (Please consider whether others in the research community would be able to implement guidelines or recommendations and/or constructively engage in the debate)

Partly

Does the article adequately reference differing views and opinions?

Yes

Are all factual statements correct, and are statements and arguments made adequately supported by citations?

Partly

Is the rationale for the Open Letter provided in sufficient detail? (Please consider whether existing challenges in the field are outlined clearly and whether the purpose of the letter is explained)

Partly

Is the Open Letter written in accessible language? (Please consider whether all subject-specific terms, concepts and abbreviations are explained)

Yes

Reviewer Expertise:

I have expertise in Circular Bioeconomy (with a global perspective) and Business models for a Circular Bioeconomy, especially related to agriculture and livestock systems.

I confirm that I have read this submission and believe that I have an appropriate level of expertise to confirm that it is of an acceptable scientific standard, however I have significant reservations, as outlined above.

# Reviewer report: Reviewer response for version 1

**DOI:** 10.21956/openreseurope.17519.r38138

**Published:** 2024-03-12

**Authors:** Rasa Pakeltiene

**Affiliations:** 1 Vytautas Magnus University, Kaunas, Lithuania

The article aims to explain the transition of the bioeconomy towards a knowledge-based sustainable system which includes the driving and restraining factors of all biobased sectors in Poland. The concept of a systemic approach to bioeconomy in Poland is explained well, clearly describing strengths and weaknesses and identifying the gaps in bio-based sectors that require some specific strategic interventions. The comprehensive strategy not only highlights the nation's commitment to sustainable development but also underscores the importance of integrating various sectors to harness the full potential of bioresources. The emphasis on a systemic approach reflects a forward-thinking mindset, fostering collaboration between government and policy actions, industries, and research institutions. Kudos to Poland for embracing such a holistic and well-articulated approach to bioeconomy.

Where applicable, are recommendations and next steps explained clearly for others to follow? (Please consider whether others in the research community would be able to implement guidelines or recommendations and/or constructively engage in the debate)

Yes

Does the article adequately reference differing views and opinions?

Yes

Are all factual statements correct, and are statements and arguments made adequately supported by citations?

Yes

Is the rationale for the Open Letter provided in sufficient detail? (Please consider whether existing challenges in the field are outlined clearly and whether the purpose of the letter is explained)

Yes

Is the Open Letter written in accessible language? (Please consider whether all subject-specific terms, concepts and abbreviations are explained)

Yes

Reviewer Expertise:

Circular Bioeconomy, EU policy, Knowledge-based rural development

I confirm that I have read this submission and believe that I have an appropriate level of expertise to confirm that it is of an acceptable scientific standard.

# Reviewer report: Reviewer response for version 1

**DOI:** 10.21956/openreseurope.17519.r36947

**Published:** 2024-03-12

**Authors:** Vlada Vitunskiene

**Affiliations:** 1 Faculty of Bioeconomy Development, Vytautas Magnus University, Kaunas, Lithuania

The article was carried out in the international project BIOEASTsUP H2020 and presents a vision of a strategic approach for the development of a national bioeconomy strategy in Poland, which can become a starting point for a wider discussion of policymakers, stakeholders and public representatives on the strategically oriented development of a knowledge-based circular bioeconomy. The authors aim to initiate the transition of the bioeconomy towards a knowledge-based sustainable system, covering the main biological sectors closely related to agriculture in the context of the European Green Deal. The article focuses on the sustainable development of niche bioeconomy sectors, for which the concept of bioeconomy imposes a double burden - to provide food for a growing population and biomass that will be used for products and processes. The focus is on three niche sectors such as organic farming, biogas and biomethane, and inland aquaculture.

 The article shows possible strategic actions in these areas, i.e. market intervention, development of research, innovation and education, and management and policy actions that could strengthen the link between the main and niche sub-sectors of the bioeconomy according to a bioeconomy strategy or action plan that will benefit from the added value of the products of the bioeconomy sectors and the increasing number of new jobs.

 The work was carried out very carefully, empirical data analysis and SWOT analysis findings were complemented by a literature review. As a result, the article will be of interest not only to policymakers and stakeholders but also to academia.

 I would suggest the authors correct the title of the article - not to use the word "paper". I would also suggest indicating the research methods in the introduction, including the SWOT analysis since the article is prepared based on the result of the analysis of the strengths, weaknesses, opportunities and threats of the Polish bioeconomy sectors.

Where applicable, are recommendations and next steps explained clearly for others to follow? (Please consider whether others in the research community would be able to implement guidelines or recommendations and/or constructively engage in the debate)

Yes

Does the article adequately reference differing views and opinions?

Yes

Are all factual statements correct, and are statements and arguments made adequately supported by citations?

Yes

Is the rationale for the Open Letter provided in sufficient detail? (Please consider whether existing challenges in the field are outlined clearly and whether the purpose of the letter is explained)

Yes

Is the Open Letter written in accessible language? (Please consider whether all subject-specific terms, concepts and abbreviations are explained)

Yes

Reviewer Expertise:

Development of a circular and sustainable bioeconomy, assessing the sustainable potential of biomass, competitiveness and sustainability of agriculture, agricultural and rural development policy

I confirm that I have read this submission and believe that I have an appropriate level of expertise to confirm that it is of an acceptable scientific standard.

# Reviewer report: Reviewer response for version 1

**DOI:** 10.21956/openreseurope.17519.r36940

**Published:** 2024-02-21

**Authors:** Efstratios Loizou

**Affiliations:** 1 Department of Regional Development and Cross Border Studies, University of Western Macedonia, Kozani, Greece

The current manuscript aims to examine a very interesting issue, that has attracted the interest of both scholars and policy makers in the last years. Generally, Bioeconomy issues and especially the design of strategies to boost Bioeconomy in every country, is a key research target.

 The paper is engaged with such Bioeconomy issues for Poland. Using the outcomes of the BIOEASTsUP project they propose how to develop bioeconomy sectors towards sustainable development. The manuscript through a SWOT analysis presents important outcomes regarding the strengths and weaknesses of Bioeconomy in Poland. Moreover, interesting is the division of bioeconomy into conventional and niche sectors and the identification of the drivers for bioeconomy development. Though, a short concept paper, the authors outline interesting conclusions regarding strategic actions for bioeconomy development, grouped into three areas.

 Apart from finding the subject of the current manuscript very interesting, its structure and language use are sufficient and satisfactory towards analyzing the issue under examination. The literature is satisfactory as basic and seminal studies are referenced.

Where applicable, are recommendations and next steps explained clearly for others to follow? (Please consider whether others in the research community would be able to implement guidelines or recommendations and/or constructively engage in the debate)

Yes

Does the article adequately reference differing views and opinions?

Yes

Are all factual statements correct, and are statements and arguments made adequately supported by citations?

Yes

Is the rationale for the Open Letter provided in sufficient detail? (Please consider whether existing challenges in the field are outlined clearly and whether the purpose of the letter is explained)

Yes

Is the Open Letter written in accessible language? (Please consider whether all subject-specific terms, concepts and abbreviations are explained)

Yes

Reviewer Expertise:

Rural and Regional Development; Bioeconomy; Input-Output Modeling; Agri-food sector economics.

I confirm that I have read this submission and believe that I have an appropriate level of expertise to confirm that it is of an acceptable scientific standard.
